# Pharmacokinetics of hyaluronidase-facilitated subcutaneous immunoglobulin 10% in pediatric patients with primary immunodeficiency disease

**DOI:** 10.1093/immadv/ltag003

**Published:** 2026-04-03

**Authors:** Zhaoyang Li, Yang Teng, Sharon Russo-Schwarzbaum, Barbara McCoy

**Affiliations:** Clinical Pharmacology & Early Clinical Development, Takeda Development Center Americas, Inc., Cambridge, MA, United States; Data Science, Safety & Regulatory, IQVIA, Wayne, PA, United States; Global Clinical Sciences, Baxalta Innovations GmbH, a Takeda Company, Vienna, Austria; Clinical Medicine, Baxalta Innovations GmbH, a Takeda Company, Vienna, Austria

**Keywords:** hyaluronidase-facilitated subcutaneous immunoglobulin 10%, primary immunodeficiency disease, pharmacokinetics, inborn errors of immunity, pediatric patients, Ig dosing strategy

## Abstract

**Introduction:**

Hyaluronidase-facilitated subcutaneous immunoglobulin 10% (fSCIG 10%) is a unique and effective treatment for primary immunodeficiency disease (PID), allowing for less frequent administration than conventional subcutaneous immunoglobulin therapies.

**Methods:**

The pharmacokinetics (PK) of fSCIG 10% in pediatric patients (aged 2 to <16 years at screening) with PID was assessed in a prospective, phase 3, open-label, multicenter, clinical trial conducted in the USA. Patients previously treated with intravenous or conventional subcutaneous immunoglobulin therapy (consistent dose for ≥3 months) received fSCIG 10% via a dose ramp-up schedule for ≤6 weeks (Epoch 1), then at full target dose every 3–4 weeks for ≤3 years (Epoch 2). Serum total immunoglobulin G (IgG) trough levels were reported throughout Epoch 2 across pediatric age groups (2 to <6, 6 to <12, and 12 to ≤16 years); serial PK were characterized at the Month 6 infusion. In total, 44 patients (mean age: 9.0 years; 59% male and 91% White) were eligible for the study.

**Results:**

Mean total IgG trough levels during Epoch 2 were similar across age groups. Geometric mean area under the IgG concentration–time curve per week ranged from 63.4 to 76.8 g·day/L, and body weight-adjusted apparent clearance ranged from 1.5 to 1.9 ml/day/kg.

**Conclusion:**

This study showed that serum total IgG trough levels were effectively maintained in pediatric patients with PID treated with fSCIG 10%, regardless of age. The dosing strategy for pediatric patients should be informed by assessing individual IgG levels and clinical status as for adults.

**Clinical trials registration:**

The study is registered with the ClinicalTrials.gov registry at https://clinicaltrials.gov/ct2/study/NCT03277313 (ClinicalTrials.gov Identifier: NCT03277313).

## Graphical Abstract

**Graphical Abstract:**
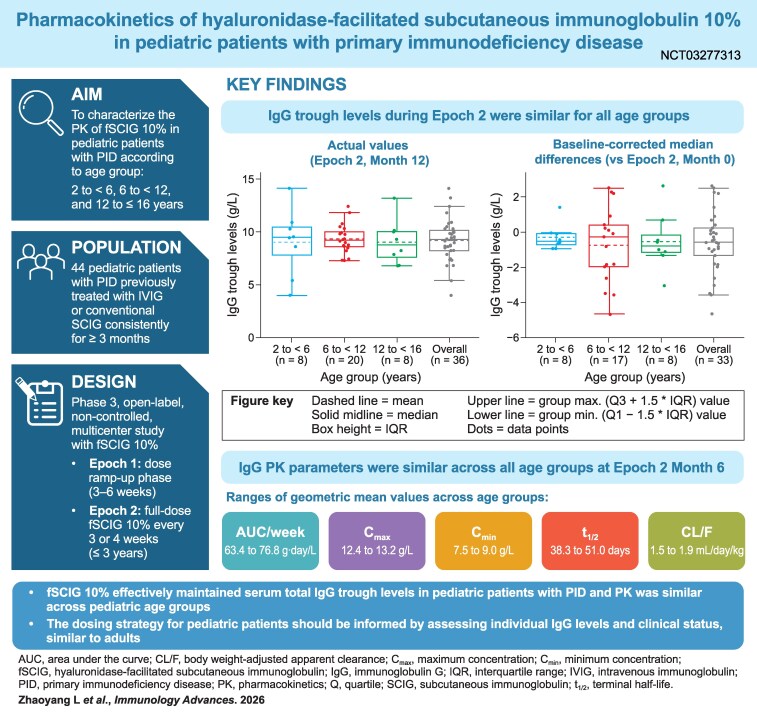

## Introduction

Primary immunodeficiency diseases (PIDs), also referred to as inborn errors of immunity, are a group of more than 550 heterogeneous clinical conditions characterized by immune system abnormalities involving more than 508 genes [1–3]. In a pediatric patient database in the USA, the prevalence of PID was 127 per 100 000 patients in 2012 [4]. The clinical presentation of patients with PIDs is variable, but includes increased susceptibility to infection, autoimmunity, allergy, bone marrow failure, and/or malignancy [2, 3]. Broadly, PIDs can be classified into disorders of adaptive immunity (abnormalities in T- and B-cell function) and disorders of innate immunity [2]. For patients with PIDs and a disorder of the adaptive immune system involving impaired production of protective antibodies, immunoglobulin (Ig) G replacement therapy (IgRT) is considered the standard of care [5–7].

Intravenous infusion allows for the delivery of higher doses of IgG with fewer local reactions than the previously more common intramuscular route [5, 8]. Nevertheless, intravenous immunoglobulin (IVIG) can be associated with systemic adverse reactions, including headache, fever, sinus tenderness, cough, myalgia, and malaise [5, 8, 9]. IVIG therapy also requires skilled personnel to establish repeated venous access, which can be challenging in pediatric patients with small veins; visits to hospitals or infusion centers, or scheduling of home infusion nurse visits, may place additional burden on patients’ quality of life [6, 9, 10]. Subcutaneous immunoglobulin (SCIG) offers an alternative treatment to IVIG [5, 8]. SCIG infusion is associated with fewer systemic adverse events than IVIG and may be administered at home by the patient or the patient’s caregiver [5, 6, 8]. However, the maximum volume and dose that can be administered in a single infusion is lower with SCIG than with IVIG, and therefore more frequent administration, with multiple infusion sites, is required [7].

Hyaluronidase-facilitated SCIG 10% [fSCIG 10% (HyQvia/HYQVIA; Baxalta Innovations GmbH/Baxalta US, Inc., both Takeda companies)], comprising a dual vial unit of IgG and recombinant human hyaluronidase (rHuPH20), is a unique SCIG therapy that combines the benefits of IVIG and conventional SCIG [6, 11]. rHuPH20 depolymerizes hyaluronan in the extracellular matrix, which transiently increases the permeability of subcutaneous tissue, thereby improving the dispersion and absorption of IgG [6, 12]. rHuPH20 permits administration of IgG in volumes similar to those with IVIG and with fewer infusion sites than conventional SCIG [10, 13]. fSCIG 10% can be administered at home at reduced dosing frequencies compared with SCIG (every 3 or 4 weeks vs daily to every 2 weeks) [6, 9, 11, 14]. As measured by the area under the concentration–time curve, fSCIG 10% provided exposure comparable to that with IVIG while resulting in a lower and slower median peak serum IgG level than IVIG [6, 15]. fSCIG 10% is approved in North America for the treatment of PID in adults and pediatric patients (aged ≥ 2 years) and as a maintenance therapy for adults with chronic inflammatory demyelinating polyradiculoneuropathy (CIDP) [11, 16]. In the European Union, fSCIG 10% is indicated as an IgRT for adults and pediatric patients (aged 0 years and above) with PID or secondary immunodeficiency disease, and as a maintenance therapy for CIDP after stabilization with IVIG [14]. In Japan, fSCIG 10% was first approved in December 2024 for the treatment of agammaglobulinemia and hypogammaglobulinemia in adults and pediatric patients (aged ≥ 2 years), and in June 2025, the approval was expanded to include use for the suppression of the progression of motor function decline in CIDP (after which improvement in muscle weakness is observed) [17].

Although the pharmacokinetics (PK) of IgG has been characterized in adults with PID, PK data from pediatric patients, especially those younger than 6 years of age, are relatively scarce [18]. It is not well understood whether changes in physiology during childhood affect drug absorption, distribution, metabolism, and excretion, and thus the PK of any therapy, including Ig therapies, over time [18, 19]. Understanding the PK profile of fSCIG 10% in pediatric patients may therefore have important implications for determining the appropriate dose, volume, and infusion rate in this population. Therefore, this study analyzed and reported PK data from a phase 3 clinical trial of fSCIG 10% in pediatric patients with PIDs according to age (aged 2 to <6 years, 6 to <12 years, and 12 to ≤16 years) [20].

## Methods and materials

### Study design

This prospective, phase 3, open-label, non-controlled, multicenter study (NCT03277313) was conducted in 17 study centers across the USA. The study comprised three Epochs: Epoch 1 was a 3- to 6-week dose ramp-up phase; Epoch 2 was the full-dose treatment phase for up to 3 years with fSCIG 10% administered every 3 or 4 weeks depending on each patient’s treatment schedule prior to enrollment; Epoch 3 was a 1-year safety follow-up, if required. Patients were eligible if they were aged 2 to <16 years at screening, had received a confirmed diagnosis of a PID involving a deficiency in antibody production, had received prior IVIG or SCIG therapy at a consistent dose for ≥3 months (excluding fSCIG 10%; minimum pre-study dose between 300 and 1000 mg/kg/4 weeks), and at screening had a serum IgG trough level >5 g/L. Patients were not eligible for enrollment if they were positive at screening or had a known history of hepatitis B surface antigen, hepatitis C virus, or human immunodeficiency virus type 1/2, had an abnormal laboratory value at screening for alanine aminotransferase or aspartate aminotransferase (>2.5 times the upper limit of normal), severe neutropenia (defined as an absolute neutrophil count ≤ 500/mm^3^), ongoing history of hypersensitivity to IVIG or SCIG, or had a severe IgA deficiency (<7.0 mg/dl) with known anti-IgA antibodies and a history of hypersensitivity. Written informed consent was obtained for all patients. Enrolled patients were stratified into age groups 2 to <6, 6 to <12, and 12 to <16 years, with a minimum of six patients in each group.

In Epoch 1, patients were treated with fSCIG 10% at the study site using a dose ramp-up schedule whereby doses were incrementally increased over a period of up to 6 weeks (Supplementary Table S1) [20]. The dose and dosing frequency were dependent on pre-study therapy, dose, and treatment interval. Patients with a body weight <40 kg started treatment with an initial infusion rate of 5 ml/h/site, increasing to a maximum of 80 ml/h/site, if tolerated; patients with a body weight ≥40 kg started treatment with an initial infusion rate of 10 ml/h/site, increasing to a maximum of 240 ml/h/site, if tolerated.

After the dose ramp-up, patients were entered into Epoch 2 of the study and received fSCIG 10% at 3- or 4-week intervals for up to 3 years, at the same monthly equivalent doses as their pre-study IgRT. For patients who had received prior IVIG therapy, the dosing frequency was dependent on that of the prior treatment, and for patients who had received prior SCIG therapy, the dosing frequency was selected at the discretion of the healthcare professional and the patient. All patients in Epoch 2 started with an initial infusion rate of 10 ml/h/site, increasing to a maximum of 160 or 300 ml/h/site, if tolerated, for body weight <40 or ≥40 kg, respectively. The initial two or three infusions were administered at the study site; for subsequent infusions, at-home self-infusion was offered between the planned site visits every 3 months for patients who had been trained and deemed capable. During Epochs 1 and 2, patients were contacted by the study site 3–5 days after every infusion to follow up on any adverse events that may have occurred during or after completion of each infusion.

An additional epoch, Epoch 3, was planned to allow for a 1-year safety follow-up, if required, for patients with anti-rHuPH20 antibody titers ≥1:160 and a treatment-related serious or severe adverse event during Epoch 1 or 2. During Epoch 3, patients would receive IVIG (GAMMAGARD LIQUID; Baxalta US, Inc., a Takeda company) and be monitored for anti-rHuPH20 titers every 3 months. However, no patient met the criteria for Epoch 3.

### Pharmacokinetics endpoints

During Epoch 2, serum total uncorrected IgG trough levels were reported at Months 0, 6, and 12. Starting at the Month 6 infusion visit, a serial PK assessment was performed. If the PK assessment was not conducted at the Month 6 visit for any reason, it was alternatively performed at the infusion visit before or after the Month 6 visit. For patients who did not undergo the PK assessments at Month 6, an additional infusion visit at the site was required. Those who received at least one dose of fSCIG 10% and had at least one available post-dose concentration data point for PK assessment, without major protocol deviations or events affecting PK analyses, were included. Patients were excluded from analyses if the blood drawing time was unknown and/or the concentration could not be determined; deviation from the protocol-specified drawing time window was not sufficient to exclude an observation.

Serum samples were collected at the Epoch 2 Month 6 visit before infusion (i.e. on Day 0 of PK assessment or within 1 h of infusion start time), at Day 2 (±1 day from infusion start time of Day 0; for patients ≥12 years only), Day 4 (±2 days from infusion start time of Day 0), Day 10 (±2 days), and Day 21 (±3 days; end of PK assessment for 3-week dosing frequency) or Day 28 (±3 days; end of PK assessment for 4-week dosing frequency). The following PK parameters were determined for uncorrected serum total IgG levels: pre-infusion concentration (*C*_trough_), maximum concentration (*C*_max_), exposure (area under the curve; AUC), apparent clearance (CL/F), minimum concentration (*C*_min_), terminal half-life (*t*_1/2_), and time to maximum concentration (*T*_max_). Additionally, PK parameters based on baseline-corrected total IgG concentrations for AUC, *C*_max_, and *T*_max_ were calculated. Serum total IgG PK parameters were reported overall and stratified by age at the time of the Epoch 2 Month 6 pre-infusion sampling (2 to <6 years, 6 to <12 years, and 12 to ≤16 years). The inclusion criteria required patients to be <16 years of age at screening; at the time of the PK assessment, three individuals had their 16th birthday prior to sampling, and therefore the age category for analysis was 12 to ≤16 years.

### Statistical analysis

Non-compartmental PK parametric calculations were performed using SAS® (SAS Institute, Cary, NC, USA) or Phoenix® WinNonlin® 8.3 (Certara, Princeton, NJ, USA). Six patients were excluded from total IgG *t*_1/2_ estimates because the adjusted regression coefficient for the terminal phase was <0.8. For AUC, one patient had fewer than three quantifiable total IgG concentrations and was therefore excluded from the associated descriptive statistics.

## Results

### Patient disposition

In total, 44 patients met the study eligibility criteria and received fSCIG 10%; 34 patients completed the study and 10 discontinued early. Mean (standard deviation [SD]) patient age was 9.0 (3.6) years and mean (range) weight was 37.8 (11.9–92.7) kg; 59% of patients were male and the majority were White (91%). The most frequently recorded diagnosis at baseline was common variable immunodeficiency (40.9% of patients), followed by specific antibody deficiency (36.4%) [20]. Of eligible patients with available PK data (*n* = 38), there were 8 patients aged 2 to <6 years, 21 patients aged 6 to <12 years, and 9 patients aged 12 to ≤16 years at the time of the Epoch 2 Month 6 pre-infusion sampling.

### IgG trough levels across Epoch 2 for all patients and stratified by age group

**Figure 1:**
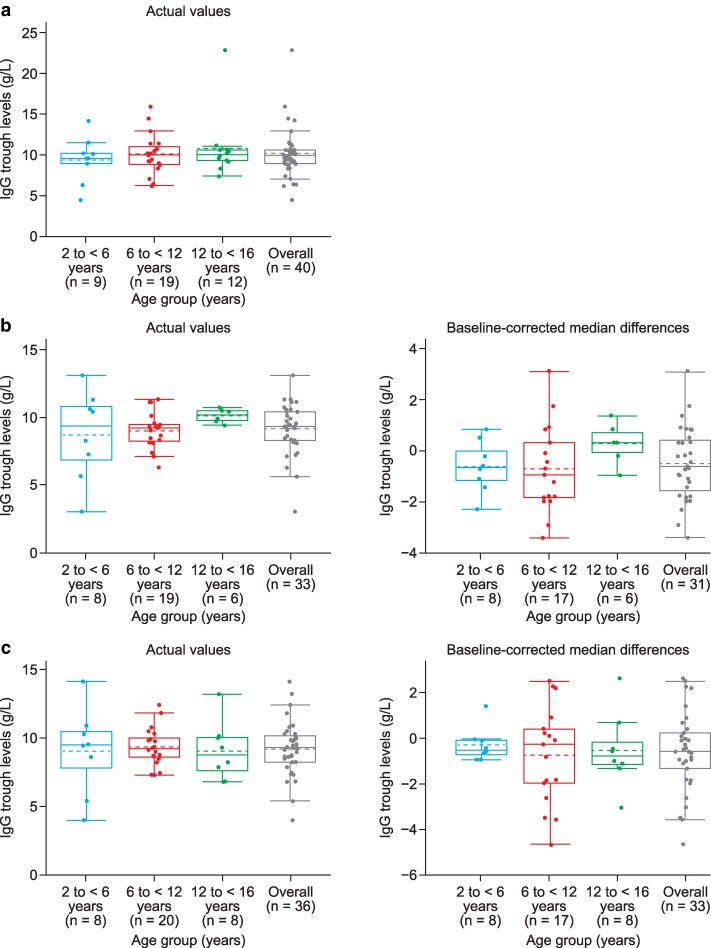
IgG trough levels by age group: (a) Epoch 2 Month 0 visit, (b) Epoch 2 Month 6 visit, and (c) Epoch 2 Month 12 visit. Age groups are based on patient age at screening. The Epoch 2 Month 0 visit was used for baseline-correction. Solid lines in the boxes represent group medians; dashed lines in the boxes represent group means; box heights represent the IQRs; upper and lower lines represent group maximum (Q3 + 1.5 * IQR) and minimum (Q1 − 1.5 * IQR) values, respectively; circles represent individual data points. IgG, immunoglobulin G; IQR, interquartile range; Q, quartile.

Mean (SD) total IgG trough levels remained stable from Epoch 2 Month 0 (10.1 [3.04] g/L) to Epoch 2 Month 6 (9.1 [1.91] g/L) and Epoch 2 Month 12 (9.2 [1.98] g/L). For all age groups, mean total IgG trough levels were similar across Epoch 2 up to Month 12, ranging from 8.7 to 9.4 g/L in the 2 to <6 years age group, from 9.0 to 10.1 g/L in the 6 to <12 years age group, and from 9.0 to 10.8 g/L in the 12 to ≤16 years age group (Fig. 1 and Table 1). The reported actual mean total trough IgG values for all pediatric patients remained above the study inclusion threshold of 5.0 g/L.

**Table 1:** IgG trough levels across Epoch 2 visits by age group.

IgG trough level (g/L)	Age group, years			Overall
	2 to <6	6 to <12	12 to ≤16	
Month 0				
Actual	*n* = 9	*n* = 19	*n* = 12	*n* = 40
Mean (SD)	9.4 (2.80)	10.1 (2.48)	10.8 (3.95)	10.1 (3.02)
Median (range)	9.6 (4.5, 14.2)	10.0 (6.2, 15.9)	10.0 (7.4, 22.9)	9.9 (4.5, 22.9)
Month 6				
Actual	*n* = 8	*n* = 19	*n* = 6	*n* = 33
Mean (SD)	8.7 (3.30)	9.0 (1.34)	10.1 (0.51)	9.1 (1.91)
Median (range)	9.3 (3.0, 13.1)	9.2 (6.3, 11.3)	10.1 (9.4, 10.7)	9.3 (3.0, 13.1)
Baseline-corrected	*n* = 8	*n* = 17	*n* = 6	*n* = 31
Mean (SD)	−0.6 (1.01)	−0.7 (1.70)	0.3 (0.81)	−0.5 (1.43)
Median (range)	−0.7 (−2.3, 0.8)	−0.9 (−3.4, 3.1)	0.3 (−1.0, 1.4)	−0.6 (−3.4, 3.1)
Month 12				
Actual	*n* = 8	*n* = 20	*n* = 8	*n* = 36
Mean (SD)	9.0 (3.15)	9.4 (1.37)	9.0 (2.11)	9.2 (1.98)
Median (range)	9.5 (4.0, 14.1)	9.2 (7.3, 12.4)	8.8 (6.8, 13.2)	9.3 (4.0, 14.1)
Baseline-corrected	*n* = 8	*n* = 17	*n* = 8	*n* = 33
Mean (SD)	−0.3 (0.76)	−0.7 (2.13)	−0.5 (1.64)	−0.6 (1.74)
Median (range)	−0.5 (−0.9, 1.4)	−0.3 (−4.7, 2.5)	−0.8 (−3.0, 2.6)	−0.6 (−4.7, 2.6)

Age groups were based on age at the time of Epoch 2 Month 6 pre-infusion PK sample; three patients had their 16th birthday prior to PK sampling and therefore, the age categorization for this subgroup analysis is 2 to <6 years, 6 to <12 years, and 12 to ≤16 years.

IgG, immunoglobulin G; SD, standard deviation.

### PK parameters for all patients and stratified by age group

**Figure 2:**
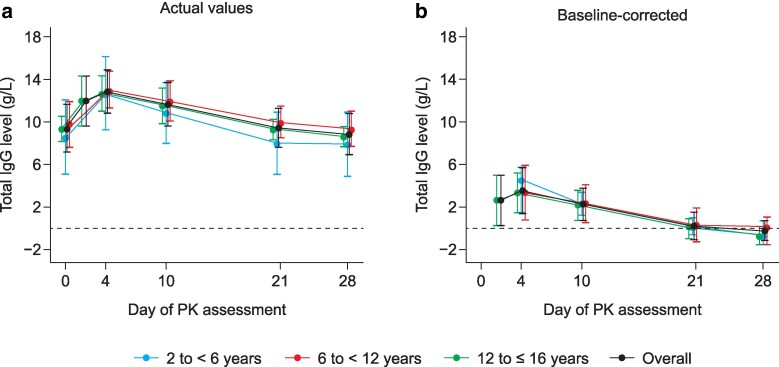
Mean serum total IgG levels by age group in Epoch 2 Month 6: (a) actual and (b) baseline-corrected values. Age groups were based on age at the time of the Epoch 2 Month 6 pre-infusion PK sample; three patients had their 16th birthday prior to PK sampling and therefore the age categorization for this subgroup analysis is 2 to <6 years, 6 to <12 years, and 12 to ≤16 years. Dashed lines indicate zero. IgG, immunoglobulin G; PK, pharmacokinetic.

At the Epoch 2 Month 6 PK assessment, the time profiles of serum total IgG levels were similar across all age groups regardless of baseline correction (Fig. 2). Across all age groups, the mean total IgG level was 9.4 g/L at Day 0 and 8.8 g/L at Day 28. Geometric mean *C*_max_ was 12.9 g/L, AUC normalized by week (AUC/week) was 74.6 g·day/L, body weight-adjusted apparent CL/F was 1.7 ml/day/kg, *C*_min_ was 8.6 g/L, *t*_1/2_ was 44.6 days, and median *T*_max_ was 5.0 days (Table 2).

**Table 2:** Descriptive statistics for serum total IgG PK parameters at the Epoch 2 Month 6 assessment.

Parameter	Age group, years						Overall (*n* = 38)	
	2 to <6 (*n* = 8)		6 to <12 (*n* = 21)		12 to ≤16 (*n* = 9)			
	Actual	Baseline-corrected	Actual	Baseline-corrected	Actual	Baseline-corrected	Actual	Baseline-corrected
*C*_max_, g/L	*n* = 8	*n* = 8	*n* = 21	*n* = 21	*n* = 9	*n* = 9	*n* = 38	*n* = 38
Geo. mean (95% CI)	12.4 (9.93, 15.43)	3.5 (2.14, 5.66)	13.2 (12.37, 14.10)	3.3 (2.45, 4.50)	12.5 (11.48, 13.69)	2.8 (1.71, 4.53)	12.9 (12.18, 13.59)	3.2 (2.62, 3.96)
Median (range)	13.9 (7.0, 15.5)	4.1 (1.0, 6.6)	13.6 (9.2, 17.5)	3.7 (0.7, 8.0)	12.5 (10.9, 15.8)	3.1 (0.9, 6.0)	13.2 (7.0, 17.5)	3.7 (0.7, 8.0)
AUC/week, g·day/L	*n* = 8	*n* = 8	*n* = 20^a^	*n* = 20^a^	*n* = 9	*n* = 9	*n* = 37^a^	*n* = 37^a^
Geo. mean (95% CI)	63.4 (51.46, 90.86)	7.2 (3.57, 14.50)	76.8 (72.49, 81.44)	9.0 (6.59, 12.21)	75.3 (69.84, 81.15)	5.8 (2.85, 11.96)	74.6 (70.55, 79.35)	7.7 (5.96, 9.96)
Median (range)	78.6 (35.4, 98.7)	9.1 (1.5, 17.6)	76.3 (57.7, 94.0)	9.8 (1.9, 21.9)	74.5 (66.4, 91.1)	5.0 (1.2, 20.0)	74.7 (35.4, 98.7)	9.1 (1.2, 21.9)
Body weight-adjusted apparent CL/F, ml/day/kg	*n* = 8	—	*n* = 20^a^	—	*n* = 9	—	*n* = 37^a^	—
Geo. mean (95% CI)	1.9 (1.26, 2.73)		1.8 (1.55, 1.97)		1.5 (1.31, 1.71)		1.7 (1.54, 1.88)	
Median (range)	1.8 (1.1, 4.8)		1.8 (1.1, 2.7)		1.5 (1.1, 2.0)		1.7 (1.1, 4.8)	
*C*_min_, g/L	*n* = 8	—	*n* = 21	—	*n* = 9	—	*n* = 38	—
Geo. mean (95% CI)	7.5 (5.12, 11.03)		9.0 (8.31, 9.65)		8.7 (7.79, 9.69)		8.6 (7.89, 9.31)	
Median (range)	8.9 (3.0, 12.4)		8.8 (6.3, 11.9)		8.9 (6.6, 10.5)		8.9 (3.0, 12.4)	
*t*_1/2_, day	*n* = 5	—	*n* = 9	—	*n* = 3	—	*n* = 17^b^	—
Geo. mean (95% CI)	38.3 (19.89, 73.89)		51.0 (35.08, 73.73)		38.7 (23.11, 64.70)		44.6 (35.18, 56.52)	
Median (range)	40.7 (18.2, 77.8)		46.1 (31.8, 126.0)		36.9 (32.3, 48.5)		40.7 (18.2, 126.0)	
*T*_max_, day	*n* = 8	*n* = 8	*n* = 21	*n* = 21	*n* = 9	*n* = 9	*n* = 38	*n* = 38
Median (range)	5.1 (2.2–12.2)	5.1 (2.2–12.2)	5.1 (2.1–26.9)	5.1 (2.1–26.9)	4.1 (2.0–7.8)	4.1 (2.0–7.8)	5.0 (2.0–26.9)	5.0 (2.0–26.9)

Age groups were based on age at the time of Epoch 2 Month 6 pre-infusion PK sample; three patients had their 16th birthday prior to PK sampling and therefore, the age categorization for this subgroup analysis is 2 to <6 years, 6 to <12 years, and 12 to ≤16 years.

AUC/week, area under the dosing frequency curve, normalized by week; CI, confidence interval; *C*_max_, maximum concentration; *C*_min_, minimum concentration; *C*_trough_, Epoch 2 Month 6 pre-infusion concentration; CL/F, clearance; geo. mean, geometric mean; IgG; immunoglobulin G; *t*_1/2_, terminal half-time; *T*_max_, time to maximum concentration.

^a^One patient had fewer than three quantifiable observed total IgG concentrations and was therefore excluded from the associated descriptive statistics.

^b^Total IgG *t*_1/2_ estimates for six patients were excluded from descriptive statistics owing to an adjusted regression coefficient <0.8, and given the limited sampling scheme, *t*_1/2_ could not be calculated using best fit estimation for an additional 12 patients.

After baseline-correction of total IgG levels, trough level at Day 28 was negative (−0.2 g/L), geometric mean *C*_max_ was 3.2 g/L, AUC/week was 7.7 g·day/L, and median *T*_max_ was 5.0 days (Table 2). Other parameters could not be estimated owing to insufficient data points after baseline correction.

**Figure 3:**
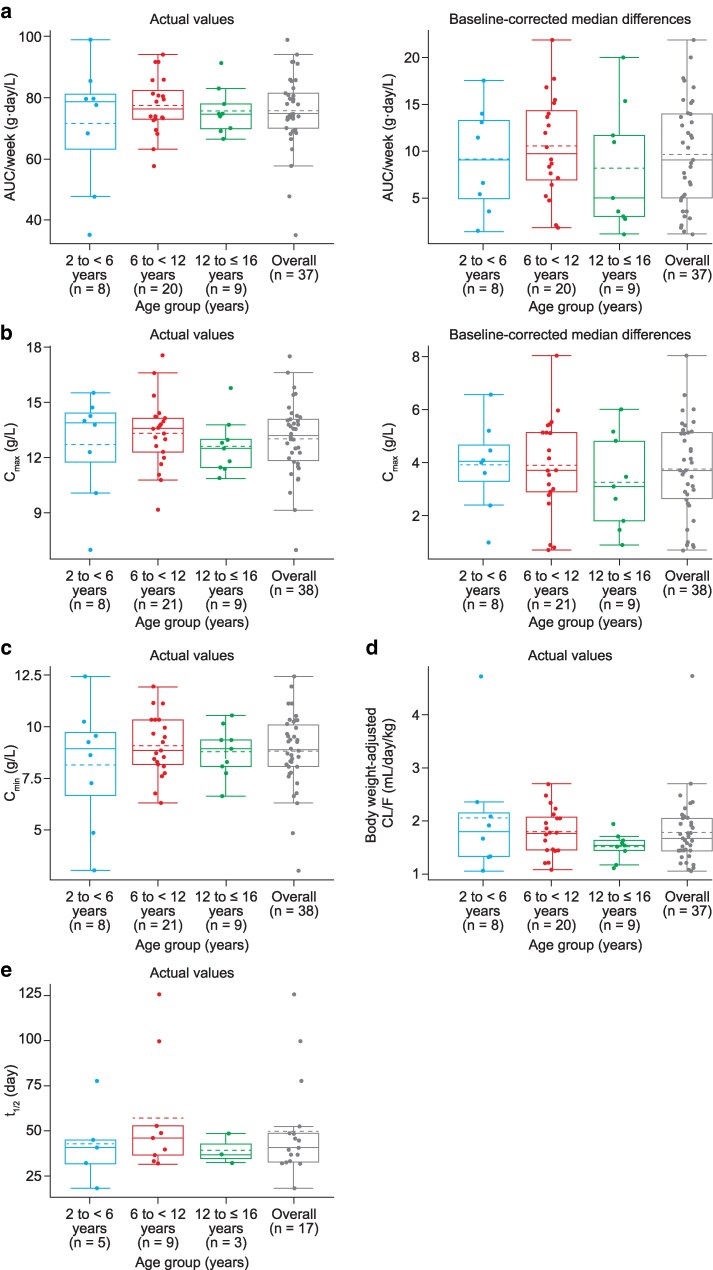
IgG PK parameters by age group at the Epoch 2 Month 6 visit: (a) AUC/week, (b) *C*_max_, (c) *C*_min_, (d) body weight-adjusted apparent CL/F, and (e) *t*_1/2_. Age groups were based on age at the time of Epoch 2 Month 6 pre-infusion PK sample; three patients had their 16th birthday prior to PK sampling and therefore the age categorization for this subgroup analysis is 2 to <6 years, 6 to <12 years, and 12 to ≤16 years. Solid lines in the boxes represent group medians; dashed lines in the boxes represent group means; box heights represent the IQRs; upper and lower lines represent group maximum (Q3 + 1.5 * IQR) and minimum (Q1 − 1.5 * IQR) values, respectively; circles represent individual data points. AUC, area under the curve; CL/F, clearance; *C*_max_, maximum concentration; *C*_min_, minimum concentration; IgG, immunoglobulin G, PK, pharmacokinetics; *t*_1/2_, half-life.

When data were stratified by age group, there were no notable differences in mean total uncorrected or baseline-corrected total IgG level between groups (Table 2, Fig. 3). Geometric mean total uncorrected *C*_max_ and AUC/week were similar across all age groups and ranged from 12.4 to 13.2 g/L and 63.4 to 76.8 g·day/L, respectively; the lowest geometric mean values for both were recorded in the 2 to <6 years age group.

The range for geometric mean uncorrected apparent CL/F normalized by body weight was 1.5–1.9 ml/day/kg across groups (Table 2). Notably, the larger range of 95% confidence intervals for the geometric mean of apparent CL/F in the 2 to <6 years age group (1.26–2.73 ml/day/kg) was owing to one patient with low exposure, with a minimum AUC/week value of 35.4 g·day/L; after removing this patient from the analysis, the apparent CL/F 95% confidence intervals were 1.06–2.36 ml/day/kg, consistent with data reported in the 6 to <12 and 12 to ≤16 years age groups (1.55–1.97 and 1.31–1.71 ml/day/kg, respectively). Additionally, geometric mean uncorrected *C*_min_ ranged from 7.5 to 9.0 g/L and was highest in the 6 to <12 years age group, and *t*_1/2_ ranged from 38.3 to 51.0 days with no apparent age-related trends. Lastly, median uncorrected *T*_max_ ranged from 5.1 days in the 2 to <6 years and 6 to <12 years age group and 4.1 days in the 12 to ≤16 years age group. With baseline correction, *T*_max_ values were unchanged.

## Discussion

This analysis reported PK parameters for serum total IgG in pediatric patients receiving fSCIG 10% in a prospective, phase 3, open-label, non-controlled, multicenter study [20]. The primary study endpoint, published previously, showed fSCIG 10% to be effective in preventing serious infections in pediatric patients with PIDs, with patients also maintaining stable and protective IgG levels across the age groups (secondary endpoint) [20]. fSCIG 10% was administered at the same dosing frequency as the patient’s prior IVIG therapy and demonstrated a favorable safety and tolerability profile with respect to both local and systemic adverse events [20].

For all patients in this analysis, geometric mean trough IgG levels remained above the study inclusion threshold of 5.0 g/L and also above the more conservative theoretical protective threshold of 7.0 g/L [21]. Additionally, across all age groups, total IgG PK parameters at the Epoch 2 Month 6 visit were similar. This finding is consistent with a recently published study, using population PK modeling and simulations based on data from eight clinical trials including pediatric and adult patients, which showed that fSCIG 10% successfully maintained IgG trough levels at or above the hypothetical protective threshold after switching from stable IVIG, irrespective of age (Table 3) [22]. Additionally, the prior modeling study showed that IgG trough levels derived from the simulations were up to 20% lower in pediatric patients than in adults, with smaller differences observed among the pediatric age groups, which is consistent with the observations made in the current study [22].

**Table 3:** Comparison of uncorrected *C*_max_ and *C*_min_ values in pediatric patients from a prior PK simulation study and this study.

	Simulation [22]^a^			This study		
	2 to <6 years (*n* = 1000)	6 to <12 years (*n* = 1000)	12 to ≤18 years (*n* = 1000)	2 to <6 years (*n* = 7)	6 to <12 years (*n* = 20)	12 to ≤16 years (*n* = 11)
*C*_max_, g/L mean (SD)	13.4 (2.0)	14.4 (2.8)	16.1 (3.2)	12.6 (3.2)	13.3 (1.8)	13.1 (2.0)
*C*_min_, g/L mean (SD)	9.3 (1.8)	10.0 (2.5)	11.0 (2.9)	7.8 (3.1)	9.2 (1.5)	8.7 (1.1)

*C*_max_, maximum concentration; *C*_min_, minimum concentration; PK, pharmacokinetic; SD, standard deviation.

^a^Steady-state PK parameters were derived from simulations of fSCIG 0.60 g/kg every 4 weeks in patients with primary immunodeficiency disease, stratified by age.

There was a slight trend of lower exposure for the 2 to <6 years age group compared with other age groups; however, this was likely driven by the data from one patient in this age group with low exposure. Despite notable inter-patient variabilities, PK parameters were generally consistent across age groups for both total and baseline-corrected total IgG at the Epoch 2 Month 6 visit. Other PK parameters, including apparent CL/F and *t*_1/2_, were similar across age groups. Overall, the data reported in the current study were also consistent with previously reported data for fSCIG 10% in pediatric patients with PID [15].

Although studies of other therapies have indicated that changes in physiology during childhood may influence drug concentration over time [19], this study found no evidence to support an age-dependent dosing adjustment in pediatric patients in addition to the current body weight-based dosing strategy. This finding is consistent with the prior population PK modeling and simulations study [22] and with a recent review that discussed five IgG therapies for PID and noted that PK parameters were similar across age groups [18]. Collectively, the evidence suggests that fSCIG 10% dose adjustments may not be warranted on the basis of age alone.

We note the limitations of this study, including the small sample size of patients in each category and the limited sampling scheme in pediatric patients, which is necessary to minimize the burden on pediatric patients. The patient population was predominantly White, and although this has also been reported in 1145 patients in the United States Immunodeficiency Network Patient Registry (72.9% White) [23], the racial background of participants in the small sample in our analysis may differ from the true distribution in the general population. Nevertheless, race was not an identified covariate for the PK of total IgG based on a population PK analysis that used a much larger dataset (*n* = 384 patients) from eight clinical studies [24]. Additionally, the study was conducted during the COVID-19 pandemic, which led to the interruption of scheduled visits. Nevertheless, this study provides valuable insights into the PK of fSCIG 10% in pediatric patients, advancing knowledge and enhancing our understanding in an area where data are scarce.

Total IgG trough levels throughout Epoch 2 remained stable for all pediatric patients aged 2 to ≤16 years of age and were maintained above the study inclusion threshold of 5.0 g/L, and also above the more conservative putative protective threshold of 7.0 g/L. PK parameters were similar across all patient age groups, and there were no clinically meaningful differences in IgG trough levels throughout Epoch 2 or at steady-state when stratified by age group. Despite the slight trends of higher apparent clearance with younger age and lower exposure in the youngest age group versus the two older groups, total IgG exposure was generally similar across age, and body weight-adjusted apparent clearance and half-life were consistent with the findings of a prior study of fSCIG 10% in patients aged at least 12 years. In conclusion, these data suggest that age has little or no effect on immunoglobulin absorption and metabolism in pediatric patients, and fSCIG 10% dose adjustments based on patient age may not be warranted. As for adults with PID, the dosing strategy for pediatric patients with PIDs should be informed by assessing individual patient IgG levels and clinical status.

## Supplementary Material

ltag003_Supplementary_Data

## Data Availability

Information pertaining to this study and the associated study protocol may be found at ClinicalTrials.gov. The datasets, including the redacted study protocol, redacted statistical analysis plan, and individual participants’ data supporting the results reported in this article, will be made available within 3 months from initial request to researchers who provide a methodologically sound proposal. The data will be provided after its de-identification, in compliance with applicable privacy laws, data protection, and requirements for consent and anonymization.
