# Supplementary material for: Pharmacokinetics of hyaluronidase-facilitated subcutaneous immunoglobulin 10% in pediatric patients with primary immunodeficiency disease
Source: Immunother Adv. 2026 Apr 3;6(1):ltag003. doi: 10.1093/immadv/ltag003 (PMC13049483; doi:10.1093/immadv/ltag003)
Supplement: ltag003_Supplementary_Data [file ltag003_supplementary_data.zip › Li et al supplementary data_12-Nov-25.docx]

# Supplementary data

## **Supplementary Table S1.** IgG dose ramp-up schedule – examples

| **Pre-study treatment** | | **Infusion doses during Epoch 1 ramp-up** | | | **Epoch 2 infusion dose and dosing frequency** |
| --- | --- | --- | --- | --- | --- |
| **Administration route** | **Dose and dosing frequency** | **Baseline infusion (followed by 1-week interval)** | **Week 1 infusion (followed by 2-week interval)** | **Week 3 infusion (followed by 3-week interval)^a^** |  |
| IVIG | 0.60 g/kg, every 3 weeks | 0.20 g/kg | 0.40 g/kg | - | 0.60 g/kg, every 3 weeks |
| IVIG | 0.60 g/kg, every 4 weeks | 0.15 g/kg | 0.30 g/kg | 0.45 g/kg | 0.60 g/kg, every 4 weeks |
| SCIG | 0.10 g/kg, every week | 0.10 g/kg | 0.20 g/kg | - | 0.30 g/kg, every 3 weeks |
| SCIG | 0.10 g/kg, every week | 0.10 g/kg | 0.20 g/kg | 0.30 g/kg | 0.40 g/kg, every 4 weeks |

The dose ramp-up period lasted for up to 6 weeks before patients entered Epoch 2 and received fSCIG 10% at the full target dose.

^a^For patients with a dosing frequency of every 4 weeks.

IgG; immunoglobulin G, IVIG; intravenous immunoglobulin; SCIG, subcutaneous immunoglobulin.
