# Supplementary material for: Pharmacokinetics of hyaluronidase-facilitated subcutaneous immunoglobulin 10% in pediatric patients with primary immunodeficiency disease
Source: Immunother Adv. 2026 Apr 3;6(1):ltag003. doi: 10.1093/immadv/ltag003 (PMC13049483; doi:10.1093/immadv/ltag003)
Supplement: ltag003_Supplementary_Data [file ltag003_supplementary_data.zip › Li et al._graphical abstract_2.00_27-Jan-26.pdf]

Pharmacokinetics of hyaluronidase-facilitated subcutaneous immunoglobulin 10% in pediatric patients with primary immunodeficiency disease

NCT03277313

AIM

To characterize the PK of fSCIG 10% in pediatric patients with PID according to age group:

2 to < 6, 6 to < 12, and 12 to ≤ 16 years

POPULATION

44 pediatric patients with PID previously treated with IVIG or conventional SCIG consistently for ≥ 3 months

DESIGN

Phase 3, open-label, non-controlled, multicenter study with fSCIG 10%

- **Epoch 1:** dose ramp-up phase (3–6 weeks)
- **Epoch 2:** full-dose fSCIG 10% every 3 or 4 weeks (≤ 3 years)

KEY FINDINGS

IgG trough levels during Epoch 2 were similar for all age groups

Actual values (Epoch 2, Month 12)

Baseline-corrected median differences (vs Epoch 2, Month 0)

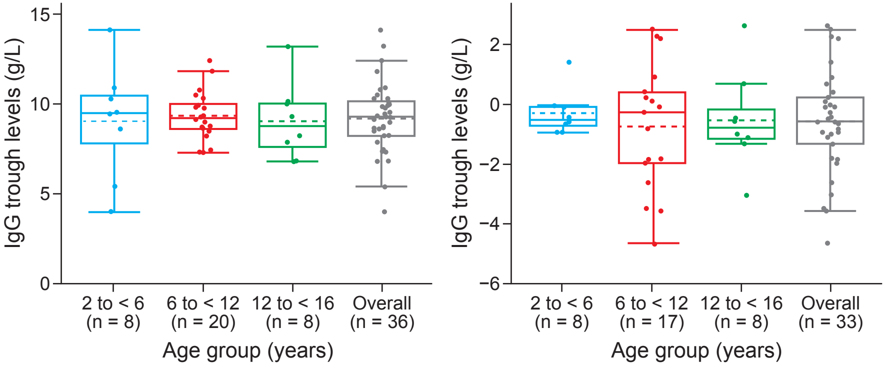

**Figure key**	Dashed line = mean	Upper line = group max. (Q3 + 1.5 * IQR) value
	Solid midline = median	Lower line = group min. (Q1 − 1.5 * IQR) value
	Box height = IQR	Dots = data points

IgG PK parameters were similar across all age groups at Epoch 2 Month 6

Ranges of geometric mean values across age groups:

AUC/week	C_{max}	C_{min}	$t_{1/2}$	CL/F
63.4 to 76.8 g·day/L	12.4 to 13.2 g/L	7.5 to 9.0 g/L	38.3 to 51.0 days	1.5 to 1.9 mL/day/kg

- **fSCIG 10% effectively maintained serum total IgG trough levels in pediatric patients with PID and PK was similar across pediatric age groups**
- **The dosing strategy for pediatric patients should be informed by assessing individual IgG levels and clinical status, similar to adults**

AUC, area under the curve; CL/F, body weight-adjusted apparent clearance; C_{max}, maximum concentration; C_{min}, minimum concentration; fSCIG, hyaluronidase-facilitated subcutaneous immunoglobulin; IgG, immunoglobulin G; IQR, interquartile range; IVIG, intravenous immunoglobulin; PID, primary immunodeficiency disease; PK, pharmacokinetics; Q, quartile; SCIG, subcutaneous immunoglobulin; $t_{1/2}$, terminal half-life.

Zhaoyang L *et al*., *Immunology Advances*. 2026
